# Hatched “egg” of thymoma with sarcoidosis

**DOI:** 10.1186/s12957-019-1696-3

**Published:** 2019-08-28

**Authors:** Tai Hato, Masatoshi Yamaguchi, Ato Sugiyama, Kohei Aoki, Yoshiaki Inoue, Hiroki Fukuda, Masatoshi Gika, Morihiro Higashi, Mitsuo Nakayama

**Affiliations:** 1 Department of General Thoracic Surgery, Saitama Medical Center, Saitama Medical University, 1981 Kamoda, Kawagoe, Saitama, Japan; 2 Department of Pathology, Saitama Medical Center, Saitama Medical University, Kawagoe, Saitama, Japan

**Keywords:** Thymoma, Round-shaped calcification, Eggshell calcification, Rim calcification, Sarcoidosis

## Abstract

**Background:**

While calcification of thymoma is common, “eggshell” calcification is rare. We report a case of an eggshell calcified thymoma that “hatched” after 4 years of follow-up. Pathologically, it revealed that sarcoidosis accompanied this case of thymoma, which might cause in calcification.

**Case presentation:**

The patient was a 68-year-old female. A 20-mm anterior mediastinal nodule completely covered with calcification was noted in an annual health check-up. However, as the nodule did not change during 6 months of follow-up, she discontinued regular examinations. Four years later, an abnormality in her chest X-ray was noted again. The tumor grew outside the calcification to reach 63 mm. She underwent resection of this anterior mediastinal tumor. Pathologically, the tumor was diagnosed as thymoma of type B1 in the WHO classification. The histology of the tumor inside and outside of the calcification was not different, suggesting that the tumor grew from the inside of the calcification. The calcification was located within the fibrotic capsule of thymoma. Sarcoidosis also presented in her lung and mediastinal lymph nodes.

**Conclusions:**

Although the mechanism of calcification of the capsule was not clear, sarcoidosis might be related to this case because macrophage accumulation and altered lipid metabolism in sarcoidosis present with similar dystrophic calcification.

## Background

Thymoma is the most common anterior mediastinal neoplasm arising in the anterior mediastinum. While calcification of thymoma is frequently observed, thymoma with “eggshell” (circumferential) calcification is very rare. Twelve cases have been reported to date [1, 2]. Here, we report a case of eggshell calcified thymoma that “hatched” after 4 years of follow-up. This case was accompanied by the swelling of multiple lymph nodes due to sarcoidosis, which is also rare.

## Case presentation

**Fig. 1:**
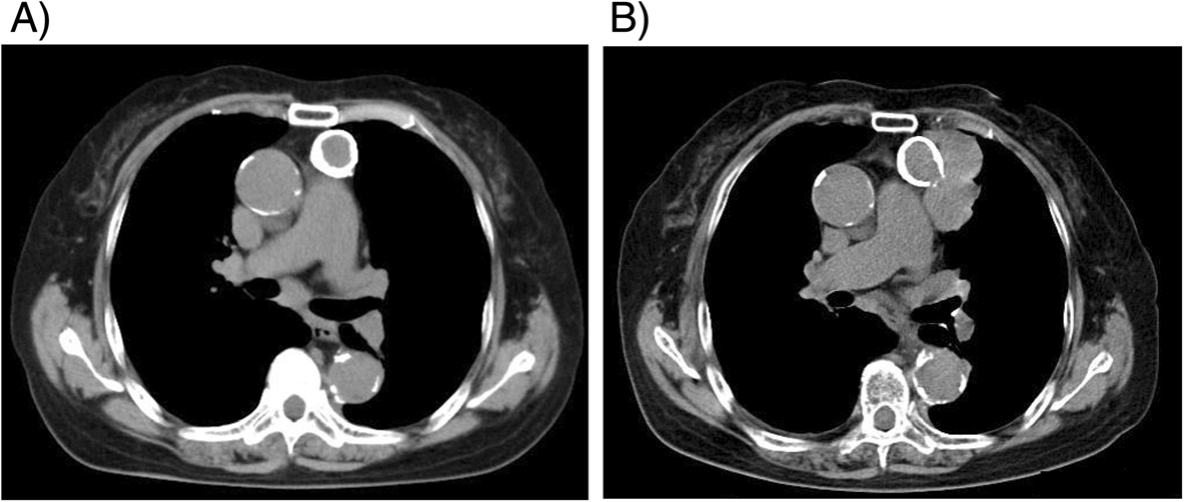
Chest computed tomography of the anterior mediastinal tumor **a** was taken when the tumor was detected for the first time. **b** Tumor growth after 4 years. The tumor grew outside the calcification and adjacent to the left lung

**Fig. 2:**
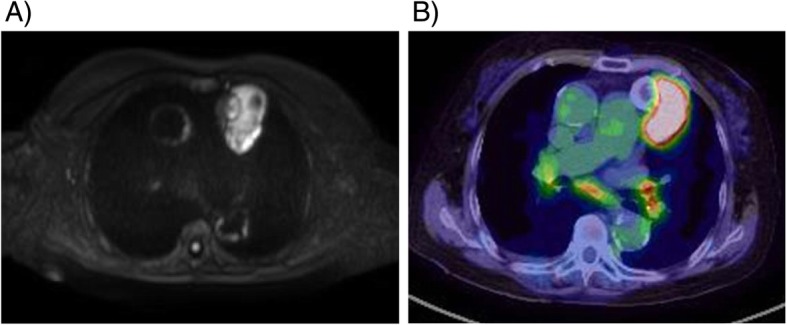
**a** Diffusion-weighted image acquired by magnetic resonance imaging. The suppression of diffusion was strong only outside the tumor. **b** 2-Deoxy-2-fluoro-D-glucose positron emission tomography of the tumor. FDG uptake was evident only outside the tumor. Tiny mediastinal lymph nodes also showed 18FDG uptake

We report the case of a 68-year-old female with an “eggshell” calcified anterior mediastinal tumor. In 2014, the anterior mediastinal tumor was discovered on a chest X-ray taken at the previous doctor. She had no symptoms. She had hypertension and chronic renal failure, but she did not undergo a detailed examination. Chest computed tomography showed a round shape; the 20-mm tumor was located in her anterior mediastinum, which was completely covered with calcification (Fig. 1a). Multiple small lymph nodes in the mediastinum and a few tiny lung nodules were also noted. Initially, the anterior mediastinal tumor was suspected as calcified lymph node swelling due to tuberculosis or silicosis. She did not undergo biopsy. By the 6-month follow-up with computed tomography, the shape and the size of the tumor did not change. She stopped visiting the clinic for examinations. Four years later, an abnormality was noted again on a chest X-ray during an annual health check-up, so she was referred to our hospital. A 63-mm, irregularly shaped anterior mediastinal tumor surrounded outside of the “eggshell” calcified lesion (Fig. 1b). The tumor showed a monotonous signal on T1- and T2-weighted magnetic resonance images. However, diffusion suppression was evident mainly outside the calcification on diffusion-weighted imaging (Fig. 2a). 2-Deoxy-2-fluoro-D-glucose (18FDG) positron emission tomography also showed glucose uptake with a maximum standardized uptake value of 6.28 only in the tumor located outside of the calcification. Middle mediastinal lymph nodes were positive for 18FDG uptake, but their size had not changed since 2014. Endobronchial ultrasonography-guided transbronchial needle biopsy of the pretracheal lymph nodes was performed, but the result was negative for tumor cells or granuloma.

**Fig. 3:**
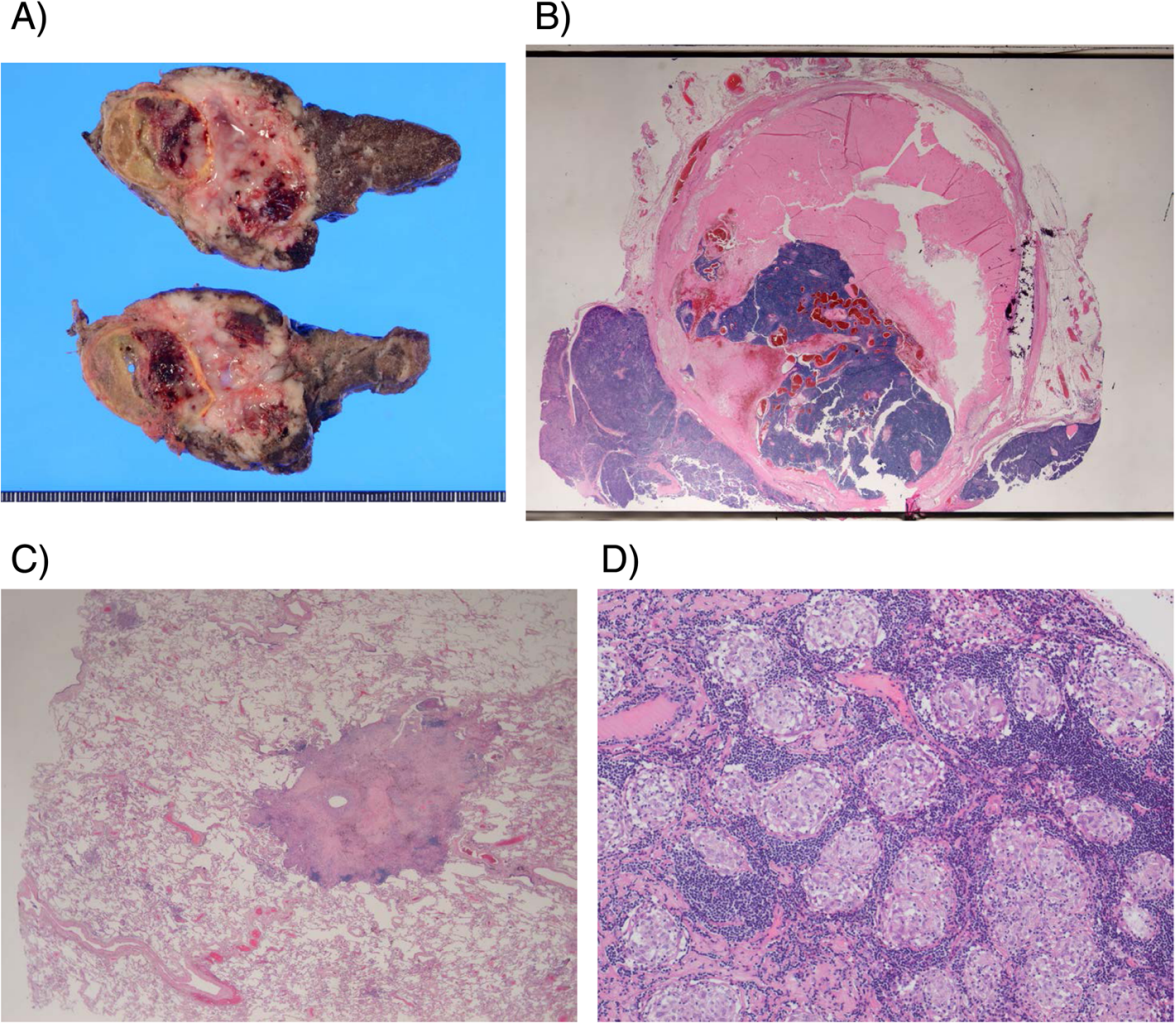
**a** Macroscopic view and **b** hematoxylin-eosin staining of the thymic tumor. The tumor was diagnosed as type B1 thymoma of WHO classification. Hematoxylin-eosin staining of **c** the lung nodule and a **d** lymph node. Small multiple granuloma formations are evident, suggesting the presence of sarcoidosis

Preoperative risk evaluation revealed that she had systemic extensive arterial sclerosis. Her right carotid artery and right femoral artery were completely obstructed due to sclerosis. The left anterior descending artery was also narrowed, and the right coronary artery was completely occluded as well. Ischemic renal dysfunction was identified. Arteriosclerotic obstruction of the lower limbs was also observed. After the risk assessment, she underwent resection of the anterior mediastinal tumor via left anterior thoracotomy. Her left lung was adhered to the tumor, so wedge resection was performed for this lesion. The postoperative course was uneventful. The tumor was diagnosed as B1-type thymoma (Fig. 3a, b). She is followed up with no additional treatment in our hospital.

Interestingly, the tumor inside and outside the calcification showed similar histology, suggesting that the tumor originated from inside the eggshell calcification. The surgical margin was negative for tumor cells. The lungs were not invaded. The final staging diagnosis of the tumor was pT1aN0M0 pStage I. Multiple epithelioid cell granulomas were identified in the resected lymph nodes and lung parenchyma. (Fig. 3c, d) There was no evidence of either mycobacterial infection or fungus by histology, suggesting the copresence of sarcoidosis.

## Discussion

While calcification of thymoma is often observed, eggshell calcification is rare. The differential diagnosis of eggshell calcification of the anterior mediastinal tumor includes thymoma, thymic carcinoma, thymolipoma, germ cell tumor, mediastinal goiter, metastatic tumor, angiosarcoma, lymphoma, asbestosis, and granulomatous disease [3–6]. Magnetic resonance imaging is helpful in terms of assessing the homogeneity/heterogeneity of the tumor. Positron emission tomography is recommended not only to assess the biological activities of the tumor but also to search for potential metastatic disease.

Twelve cases of thymoma with eggshell calcification were reported previously [1, 2]. Among them, four cases accompanied myasthenia gravis. Any type of WHO histological classification could show this type of calcification.

Nakada et al. classified them into “inner” or “outer” types according to the location of calcification [1]. Our case is unique in that we observed the course of tumor growth. The original tumor had an outer type of calcification but “hatched” later and developed into the inner type tumor.

Histologically, the eggshell calcification developed within the fibrotic capsule of the thymoma. A similar finding was reported by Sano et al. [7]. Others reported that a calcification developed inside the tumor [8]. Different locations of calcification might suggest different calcification mechanisms. The pathogenesis of calcification is not well understood for these “eggshell” types of calcification. Low et al. reported a case of outer type calcification, which was thought to be due to asbestos exposure [4]. Dystrophic calcification might be the mechanism. Dystrophic calcification can occur in the presence of degenerative tissues, necrotic tissues, entrapment of pre-existing calcified scar tissue or granulomatous tissue, and mucus-producing areas adjacent to tumor cells. It is common to see cholesterin crystals in calcification, suggesting a disorder of lipid metabolism. The copresence of sarcoidosis with thymoma is rare [9]. Sarcoidosis might enhance calcification of the fibrotic capsule in our case. Patients with sarcoidosis have been reported to develop calcification in the lung, mediastinal lymph nodes, or in the dermis. Recently, it was revealed that patients with sarcoidosis have an increased risk of arterial sclerosis due to altered lipid metabolism, increased oxidative stress, and macrophage accumulation [10]. All these components of sarcoidosis share the same inflammatory components of dystrophic calcification. Our patient also showed extensive arterial sclerosis. A similar calcification process might occur in the capsule of the thymoma, but there is no clear histological evidence to show it.

Interestingly, 18FDG uptake was negative inside the calcification. A similar finding was observed in the case of Harris et al. [11]. Due to a thick wall of calcification, the blood supply inside was limited, which can induce hypoxia in this area. This finding is also supported by the findings of diffusion-weighted magnetic resonance imaging. However, whether dystrophic calcification precedes microcirculatory disorder of the fibrotic capsule or vice versa is unclear.

## Conclusions

Here, we report a rare case of eggshell calcified thymoma with sarcoidosis. It is interesting that increased oxidative stress, alterations in lipid metabolism, and the accumulation of macrophages are reported in sarcoidosis and share similar features with dystrophic calcification. Although it might be a coincidence, sarcoidosis might be related to eggshell calcification in this case.

## Data Availability

All data generated or analyzed are included in this published article.
